# Sensory signaling mediates the systemic metabolic and neurological effects of epigallocatechin gallate

**DOI:** 10.3389/fnut.2026.1863138

**Published:** 2026-07-03

**Authors:** Yamato Yoshida, Naoki Iida, Kenshin Iwasa, Akuru Saito, Kenta Aso, Yasuyuki Fujii, Sergio Modafferi, Vittorio Calabrese, Makoto Ohmoto, Keiko Abe, Naomi Osakabe

**Affiliations:** 1 Systems Engineering and Science, Graduate School of Engineering and Science, Shibaura Institute of Technology, Saitama, Japan; 2 Central Research Institute, ITOEN Ltd., Makinohara, Japan; 3 SIT Laboratory, Shibaura Institute of Technology, Saitama, Japan; 4 Department of Applied Biological Chemistry, Graduate School of Agriculture, Osaka Metropolitan University, Osaka, Japan; 5 Department of Biomedical and Biotechnological Sciences, University of Catania, Catania, Italy; 6 Department of Physical Therapy, Faculty of Health Care, Takasaki University of Health and Welfare, Takasaki, Japan; 7 Department of Applied Biological Chemistry, Graduate School of Agricultural and Life Sciences, The University of Tokyo, Bunkyo City, Japan

**Keywords:** bitter taste receptor, epigallocatechin gallate, glucose tolerance, sensory nutrition, skeletal muscle hypertrophy

## Abstract

**Background:**

Epigallocatechin gallate (EGCG), the primary green tea flavanol, is renowned for its diverse health benefits; however, its low systemic bioavailability presents a long-standing paradox in nutritional science. We hypothesized that EGCG exerts its physiological effects via oral chemosensory signaling pathways, independent of intestinal absorption.

**Methods:**

This study utilized wild-type and Skn-1a^−/−^ mice (lacking bitter taste signaling) to evaluate acute metabolic responses. Furthermore, a chronic study using high-fat/high-sucrose diet (HFSD)-fed rats was conducted to investigate the long-term effects of EGCG on systemic metabolism, neuroinflammation, and adipose and skeletal muscle morphology.

**Results:**

Acute oral administration of EGCG or the bitter tastant denatonium benzoate significantly attenuated glycemic excursions and elevated plasma glucagon-like peptide-1 (GLP-1) levels in wild-type mice. Crucially, these effects were completely abolished in Skn-1a^−/−^ mice, identifying bitter taste receptors as essential mediators. In the chronic rat model, repeated oral EGCG treatment effectively reduced food intake, body weight gain, and adiposity. Beyond metabolic regulation, EGCG suppressed Iba-1 expression in the hippocampal dentate gyrus, indicating an anti-neuroinflammatory effect. Notably, EGCG increased the cross-sectional area of both the soleus and extensor digitorum longus muscles across all diet groups, mimicking the beneficial effects of physical exercise.

**Conclusion:**

These findings indicate that EGCG acts as a “metabolic trigger” through Skn-1a-dependent chemosensory pathways, primarily involving T2R signaling. By demonstrating that bitter-related chemosensory signaling regulates systemic homeostasis and provides neuroprotective effects, this study supports a new concept of “sensory nutrition.” This research positions gastrointestinal and oral chemosensors as a novel and non-invasive therapeutic target for managing metabolic syndrome and cognitive decline, overcoming the limitations of systemic bioavailability.

## 1 Introduction

The health benefits of bitter compounds found in foods and herbal medicines are well-documented; however, the precise mechanisms underlying their systemic effects remain largely elusive. It has been hypothesized that these effects are mediated by taste stimulation and subsequent physiological signaling, but the specific details require further investigation.

Central to this investigation are bitter taste receptors (T2Rs or Tas2Rs), which are G protein-coupled receptors (GPCRs) primarily identified in type II taste receptor cells within taste buds (1–4). Interestingly, recent studies have identified “extra-gustatory” T2Rs in various extra-oral tissues, including the brain (5), the heart (6), the respiratory system (7), the reproductive system (8), the bone marrow stroma, and the vascular walls (9). The widespread distribution of these receptors suggests their fundamental role in maintaining systemic homeostasis far beyond simple taste perception.

Of particular interest is the functional relationship between T2Rs in the digestive tract and gastrointestinal hormones secreted by enteroendocrine cells (EECs) (10, 11). There is an expanding consensus that gut hormones, secreted in response to bitter, sweet, and other sapid compounds, regulate glucose tolerance and energy homeostasis, rendering them promising therapeutic targets for metabolic disorders (12). Recent findings have further refined this model, revealing that a subset of EECs, termed “neuropod cells,” establish direct synaptic connections with nerves in the mucosa of the small intestine and colon (13). These cells synapse with vagal ganglion neurons and secrete glutamate as a neurotransmitter to rapidly transmit sensory information from the gut to the brain (13, 14). Following this signaling axis, preproglucagon (PPG) neurons in the nucleus tractus solitarius (NTS) have been reported to be activated by vagal nerve stimulation, leading to the inhibition of gastrointestinal motility and appetite (15). This gut-brain pathway, potentially enhanced by solitary chemosensory cells (SCCs), represents a potent regulatory mechanism for dietary compounds.

Among these compounds, polyphenols represent a highly diverse class of plant secondary metabolites characterized by multiple hydroxyl groups on their aromatic rings, with over 8,000 structural variants identified to date (16). Many polyphenols are perceived as bitter (17, 18). Notably, recent literature has reinforced this sensory-mediated perspective, highlighting that green tea flavanols, such as epicatechin and epigallocatechin, act as direct ligands for specific bitter taste receptors, including T2R4, T2R14, T2R39, and T2R43 (19) Gallate-type catechins found in green tea, such as epigallocatechin gallate (EGCG), are particularly reported for their potent bitterness and astringency (20, 21). In addition, our recent study involving young panelists confirmed that EGCG exhibits astringency along with a strong bitter taste (22). Numerous intervention studies have demonstrated that acute or repeated intake of green tea catechins can suppress postprandial blood glucose levels during glucose tolerance tests (23, 24). Conversely, the underlying mechanism of action for these gallate-type catechins remains enigmatic, as they exhibit poor systemic bioavailability and low absorption into the bloodstream (25, 26).

This “bioavailability paradox” strongly implies that these substances exert their biological activities via interaction with sensory receptors in the gastrointestinal tract, rather than through systemic circulation.

Addressing this discrepancy, the present study elucidates a molecular mechanism through which EGCG-induced sensory signals, rather than its absorbed metabolites, directly regulate systemic homeostasis. We investigated whether the taste signaling of epigallocatechin gallate (EGCG) contributes to its health-promoting effects, including improved glucose tolerance. First, we performed an intraperitoneal glucose tolerance test (IPGTT) in wild-type mice following oral administration of EGCG, using the typical bitter substance denatonium benzoate (DB) as a highly selective T2R agonist and positive control. To verify the necessity of the bitter taste signaling pathway, we employed Skn-1a (Pou2f3)^−/−^ mice, which lack the differentiation of chemosensory cells required for taste transduction. Skn-1a is a master transcription factor essential for the differentiation of type II taste cells, which exclusively express G-protein-coupled receptors for bitter (T2Rs), sweet, and umami tastes. Notably, these mice lack Transient receptor potential channel M5 (Trpm5)-expressing cells not only in the oral cavity but throughout the gastrointestinal tract, effectively abolishing the chemosensory transduction required for these taste modalities (27). By using denatonium benzoate (DB) alongside EGCG, this model allows us to isolate the contribution of the T2R-mediated signaling axis from other non-sensory metabolic pathways. Furthermore, to evaluate chronic effects on appetite and systemic metabolism, rats were fed either a standard diet or a high-fat, high-sucrose diet (HFSD) with oral EGCG for 6 weeks, allowing for the assessment of alterations in glucose tolerance and tissue-specific changes in adipose tissue, skeletal muscle, and the brain.

## 2 Materials and methods

### 2.1 Materials

Epigallocatechin gallate (EGCG) in green tea leaves was manufactured from ITOEN Ltd. (Shizuoka, Japan). Denatonium benzoate (DB) was purchased from Fujifilm Wako Pure Chemical Corporation (042-23563, Tokyo, Japan).

### 2.2 Animals and diets

Eight-week-old male C57BL/6J mice were obtained from CLEA Japan, Inc. (Tokyo, Japan). Skn-1a^−/−^ mice with a C57BL/6J congenic background (i.e., B6.129-Pou2f3tm1Abek) were generated as previously described 20 and maintained by crossing homozygous mice; these mice were used at 9 weeks of age. We used Skn-1a^−/−^ mice, which were backcrossed onto a C57BL/6J background for at least eight generations. Skn-1a is a critical transcription factor required for the differentiation of type II taste cells, which harbor G-protein-coupled receptors for bitter, sweet, and umami tastes. Notably, Skn-1a is also essential for the development of solitary chemosensory cells (SCCs) in extra-oral tissues, including the gastrointestinal tract. Therefore, Skn-1a^−/−^ mice serve as an ideal model lacking systemic bitter taste signaling.

Male Wistar rats (11 weeks old, body weight 370–390 g) were obtained from Saitama Experimental Animal Supply (Tokyo, Japan). During the 1-week acclimation period, the animals were carefully handled to minimize stress. The animals were housed at controlled room temperature (24–26 °C) under a 12-h light/dark cycle (light cycle: 7:00–19:00, dark cycle: 19:00–7:00) with *ad libitum* access to water and food. The control diet (CD, MF^®^) for laboratory animals was obtained from Oriental Yeast Co., Ltd. (Tokyo, Japan). A high-fat diet (HFD32^®^) was obtained from CLEA Japan Inc (Tokyo, Japan). In repeated treatment experiment, the standard diet (CD) group received purified water. To establish a robust model of metabolic syndrome, the high-fat diet (HFD) group was provided with 30% (w/v) sucrose in their drinking water (forming a High-Fat/High-Sucrose Diet [HFSD] model). This HFSD protocol was selected because the combination of liquid sucrose and dietary fat more effectively induces insulin resistance and visceral adiposity than HFD alone.

The study was conducted in accordance with the ARRIVE guidelines, and the experimental protocol was approved by the Animal Experimentation Committee of Shibaura Institute of Technology (approval number: AEA 24014). All animals were humanely raised according to the guidelines. The primary objective of this study was to detect differences in the response to EGCG-mediated bitterness between wild-type and Skn-1a^−/−^ mice; thus, male mice of comparable ages were used. Additionally, since individual housing of mice can induce significant social isolation stress and potentially confound metabolic data (28), it is challenging to obtain reliable measurements of individual food intakes. Therefore, male rats were employed for the chronic study, as they are more resilient to individual housing, allowing for the precise monitoring of food consumption and the evaluation of the long-term effects of repeated EGCG administration.

### 2.3 Intraperitoneal glucose tolerance test (IGPPT) following a single oral dose of DB or EGCG

#### 2.3.1 Experimental procedure

C57BL/6J mice or Skn-1a (Pou2f3)^−/−^ mice were randomly divided into a purified water (control) group, an EGCG (100 mg/kg) group, and a DB (1 mg/kg) group. The sample size (*n* = 8 per group) was calculated using G^*^Power 3.1 to ensure a statistical power >0.80 with a Type I error rate (α) of 0.05. The dosage of the DB was determined based on previous reports (29), and the dosage of EGCG was determined by a dose-setting test (Supplementary Table 1). To ensure the translational relevance of this dosage, the Human Equivalent Dose (HED) was calculated using the FDA-recommended body surface area (BSA) scaling method (30). Based on a *Km* ratio of 3/37 (mouse to human), the murine dose of 100 mg/kg translates to an HED of approximately 8.1 mg/kg. This dose corresponds to a total daily intake of 567 mg for a 70 kg human adult.

Fasting was initiated 16 h before the experiment, followed by forced single oral administration of each test substance, and blood sampling was performed at rest. Blood glucose levels were measured prior to administration using an animal lancet (3 mm, 18311310, MEDI point, USA) inserted into the jugular vein. Thirty minutes later, D(+)-Glucose (043-311631, Fujifilm Wako Pure Chemical Corporation, Tokyo, Japan) at a dose of 1 g/kg was administered via intraperitoneal injection, and blood glucose was measured at 15, 30, 60, and 120 min post-injection. After 120 min, approximately 0.5mL of blood containing EDTA was collected. Blood glucose levels were measured using the Nipro FreeStyle Freedom Lite Blood Glucose Self-Monitoring System (30854000, Nipro Corporation, Japan).

#### 2.3.2 Measurement of blood glucagon-like peptide (GLP)−1 and insulin

Blood samples collected 120 min after glucose administration were treated with aprotinin (010-11834, Fujifilm Wako Pure Chemical Corporation, Tokyo, Japan) at 500 kIU/mL and 0.5 mM EDTA (345-01865, Fujifilm Wako Pure Chemical Corporation, Tokyo, Japan) at 10 μg/mL. After collecting, the blood was centrifuged (3,000 rpm, 4 °C, 10 min), and the plasma was collected and stored at −80 °C until measurement. Total GLP-1 was measured using the GLP-1 ELISA Kit Wako, Highly Sensitive (299-75501, Fujifilm Wako Pure Chemical Corporation, Tokyo, Japan), and insulin was measured using the LBIS™ Mouse Insulin ELISA Kit (High Sensitivity) (296-89801, Fujifilm Wako Pure Chemical Corporation, Tokyo, Japan).

### 2.4 Repeated oral administration experiment of EGCG

#### 2.4.1 Experimental procedure

Following a 2-week period of being fed a basal diet, the rats were randomly divided into two dietary groups: one group was fed a standard diet (MF^®^, Oriental Yeast Co., Ltd., Tokyo, Japan), and the other group was fed a high-fat diet (HFD32^®^, CLEA Japan Inc., Tokyo, Japan). The latter diet was formulated on the basis of the AIN-93 standard, with the fat content increased to 36.7% (Supplementary Table 2). The subjects in the HFSD group were also given a sucrose solution with a concentration of 30%. Each dietary group was further subdivided into two treatment groups receiving either distilled water (DW) or EGCG at a dose of 100 mg/kg body weight (*n* = 6 per group). The sample size (*n* = 6 per group) was calculated using G^*^Power 3.1 to ensure a statistical power >0.80 with a Type I error rate (α) of 0.05. Subsequently, body weight, food intake, and water intake were measured daily for 6 weeks.

#### 2.4.2 IGPPT following repeated oral dose of EGCG

Following a 6-week feeding period, the animals were subjected to an overnight fast, after which a IGPPT was conducted, as outlined in Section 2.3.

#### 2.4.3 Histopathological observation

Following the blood glucose measurement process, the rats were anesthetized using a combination of medetomidine (0.3 mg/kg), midazolam (4.0 mg/kg), and butorphanol (1.0 mg/kg), which were administered intraperitoneally, and then the rats were dissected.

For histopathological observation, the soleus, extensor digitorum longus (EDL), brown adipose tissue (BAT), and inguinal and epididymal adipose tissues, as well as the brain, were embedded in FSC 22 Blue (3801481; Leica Biosystems, Nussloch, Germany). The blocks were frozen using isopentane (Fujifilm Wako Pure Chemical Corporation, Osaka, Japan) on dry ice and stored at −80 °C. Adipose tissue sections (8 μm thick) were prepared using a cryostat (CM1950; Leica Biosystems) and stained with hematoxylin and eosin (HE) according to standard protocols. For ionized calcium-binding adapter molecule 1 (Iba-1) immunostaining, frozen brain sections were incubated with a blocking reagent (06349-64, Blocking One Histo; Nacalai Tesque, Inc., Kyoto, Japan) for 10 min at 4 °C. The sections were then incubated overnight at 4 °C with a rabbit anti-Iba-1 antibody (for Immunochemistry; 1:1,000, 019-19741, Fujifilm Wako). Subsequently, the sections were incubated for 1 h at 4 °C with an Alexa Fluor^®^ 488-conjugated goat anti-rabbit IgG H&L secondary antibody (1:200, ab150077; Abcam). Finally, the sections were mounted using VECTASHIELD Mounting Medium with DAPI (H-1200; Vector Laboratories, CA) for nuclear counterstaining.

The sections were observed using a digital microscope (BZ-X800, Keyence Corporation, Osaka) with Z-stack settings. Analysis was performed using the BZ-H4A analysis software (Keyence Corporation). Three sections were randomly selected from each individual, and the average of the three measurements was used as the data for that individual. Observations were conducted by three skilled technicians. BAT scoring was performed in accordance with previously reported methods (31).

Regions of interest (ROIs) in the coronal brain sections were defined using the Freehand tool in NIH ImageJ software, with reference to the Allen Brain Atlas. The proportion of Iba-1-positive cells was analyzed in hippocampal regions, including the dentate gyrus (DG), CA1, and CA3. The Iba-1-positive cell ratio was calculated by dividing the number of Iba-1-positive cells by the area of each hippocampal region, and the resulting value was used as the representative data point for each animal.

### 2.5 Statistical analysis

The sample size was determined by power analysis using G^*^Power software (version 3.1.9.7; Heinrich Heine University Düsseldorf, Germany), with a significance level (α) of 0.05 and a power (1–β) greater than 0.8. (Accessed April 8, 2025). All statistical analyses were conducted using the software GraphPad Prism 10 (https://www.graphpad.com/features). The sample size for the animal study was determined from the preliminary experiment results using a power test with a significance level of 0.05 and a power of 0.9. The data are presented as mean ± standard deviation. The normality of the samples was tested using the Shapiro–Wilk test, and the data was accepted using the Smirnov–Grubbs test. After two-way analysis of variance or Kruskal–Wallis tests, *post hoc* comparisons were performed using Tukey's multiple comparisons test, Bonferroni's multiple comparisons test, or Dunn's multiple comparisons test. The probability of *p* < 0.05 was considered significant.

## 3 Results

### 3.1 EGCG and DB improve glucose tolerance via Skn-1a-dependent signaling

**Figure 1:**
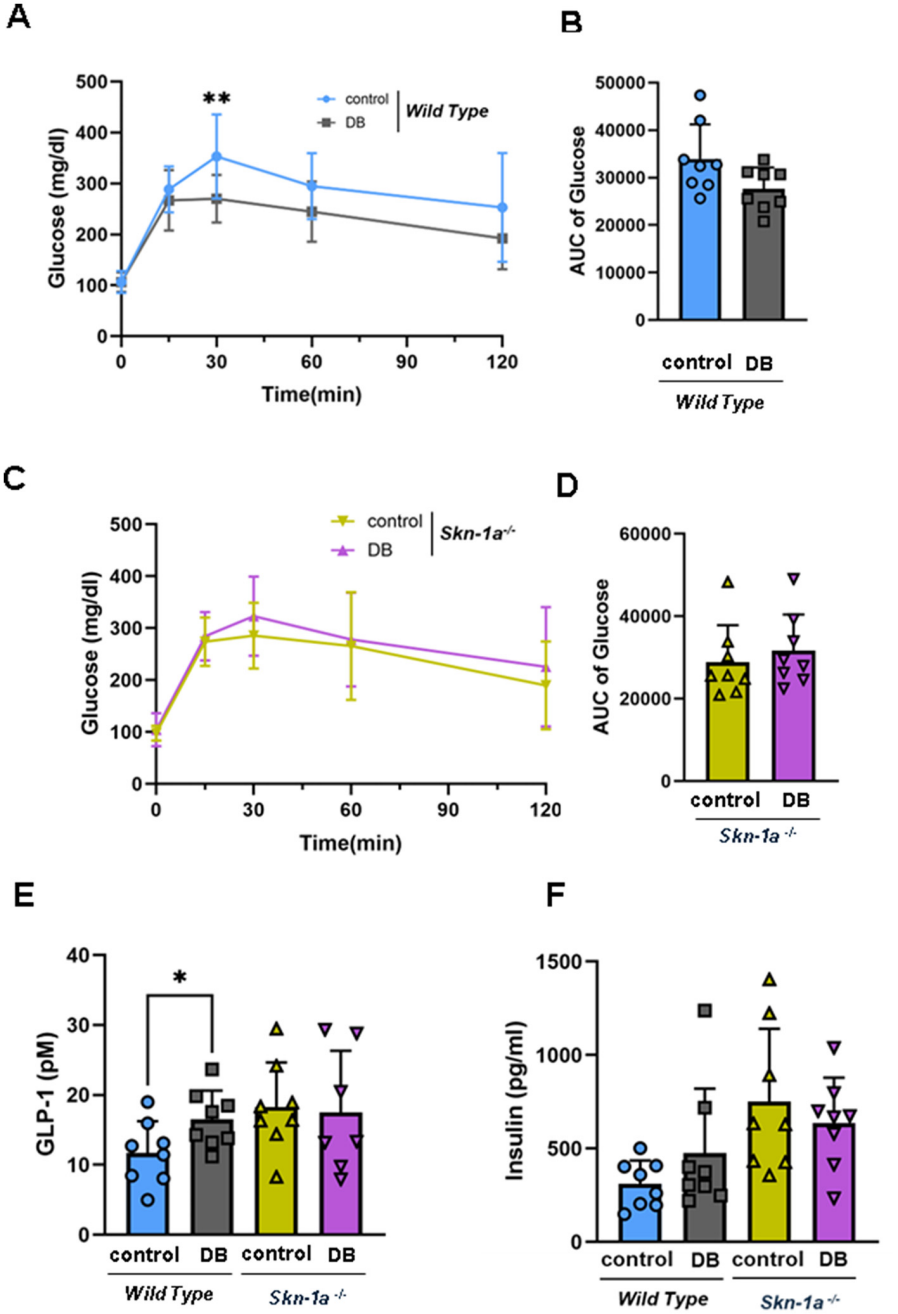
The impact of a single oral dose of denatonium benzoate (DB) on glucose tolerance, assessed via the intraperitoneal glucose tolerance test (IPGTT). Changes in blood glucose **(A)** and the area under the curve (AUC) **(B)** in wild-type mice. Changes in blood glucose **(C)** and AUC **(D)** in *Skn*−1*a*^−*/*−^ mice. Total blood GLP-1 **(E)** and insulin **(F)** concentrations 120 min after glucose administration. Values are expressed as mean ± SD (*n* = 8 per group). Statistical analyses for **(A, C)** were performed using two-way ANOVA followed by Tukey's multiple comparisons test. For **(B, D)**, an unpaired *t*-test was used. For **(E, F)**, one-way ANOVA followed by Bonferroni's multiple comparisons test was performed. Statistical significance: *p < 0.05, **p < 0.01.

Wild-type mice were orally administered DB at 1 mg/kg, and the results of IGPPT are shown in Figure 1A. Compared with the control group receiving water, pre-administration of DB significantly suppressed the elevation of blood glucose levels 30 min after administration. As shown in Figure 1B, during the 120-min observation period, the area under the curve (AUC) of blood glucose levels tended to decrease with DB administration. On the other hand, as shown in Figures 1C, D, in Skn-1a^−/−^ mice, DB did not affect blood glucose levels or AUC after the glucose tolerance test. Blood total GLP-1 and insulin concentrations 120 min after glucose administration are shown in Figures 1E, F. GLP-1 concentrations significantly increased with DB administration (Figure 1E). Serum insulin concentrations showed a tendency to rise in the DB group (Figure 1F). However, such changes were not observed in Skn-1a^−/−^ mice (Figures 1E, F).

**Figure 2:**
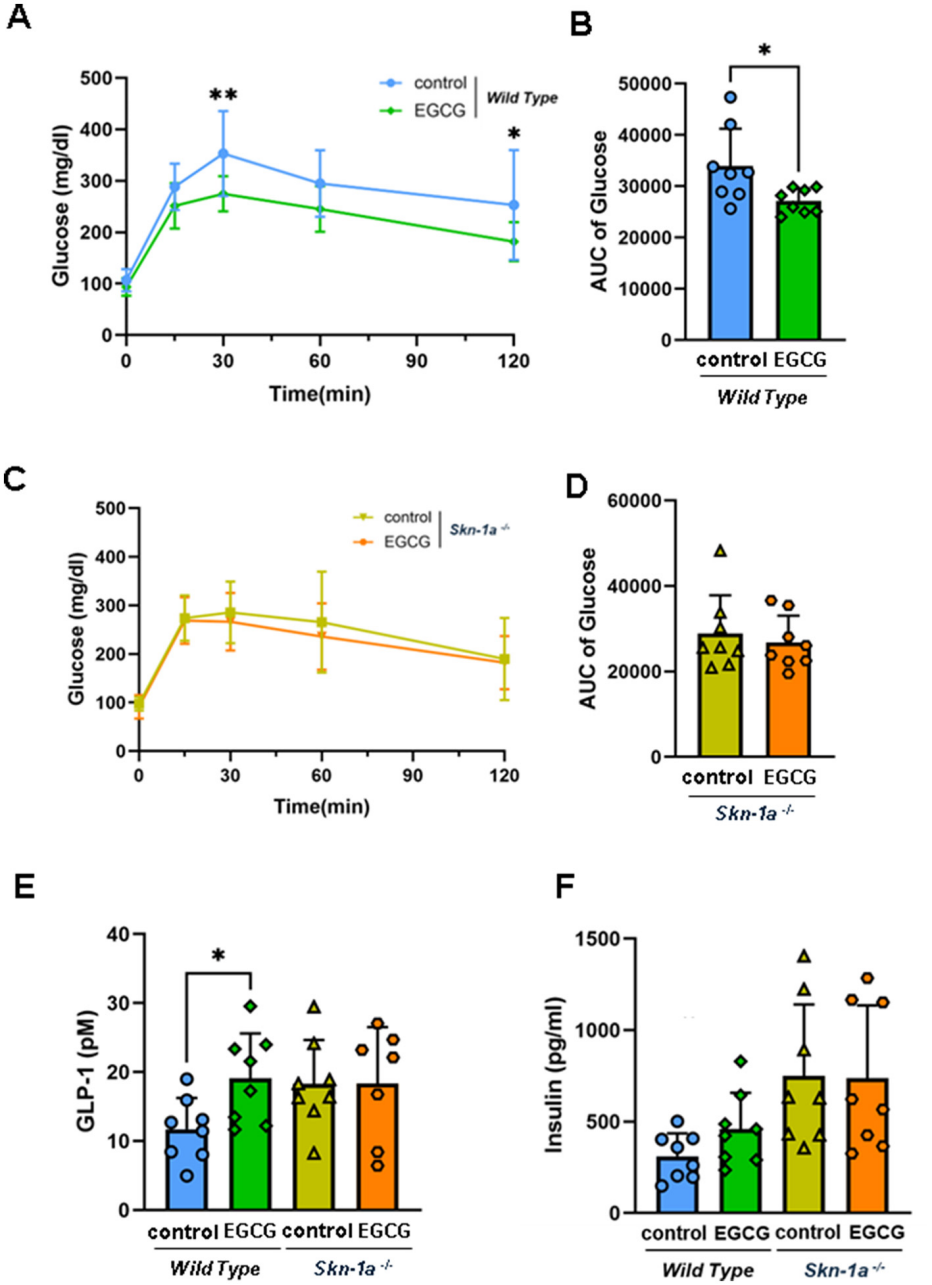
Impact of a single oral dose of denatonium benzoate (DB) on glucose tolerance, assessed via the intraperitoneal glucose tolerance test (IPGTT). Changes in blood glucose **(A)** and the area under the curve (AUC) **(B)** in wild-type mice. Changes in blood glucose **(C)** and AUC **(D)** in *Skn-1a* ^−*/*−^ mice. Total blood GLP-1 **(E)** and insulin **(F)** concentrations 120 min after glucose administration. Values are expressed as mean ± SD (*n* = 8 per group). Statistical analyses for **(A, C)** were performed using two-way ANOVA followed by Tukey's multiple comparisons test. For **(B, D)**, an unpaired *t*-test was used. For **(E, F)**, one-way ANOVA followed by Bonferroni's multiple comparisons test was performed. Statistical significance: *p < 0.05, **p < 0.01 vs. control.

Figure 2 shows the results of an IGPPT after oral administration of 100 mg/kg EGCG. In wild-type mice, the EGCG group exhibited a significant decrease in blood glucose levels and AUC at 30 min compared to the control group (Figures 2A, B). However, such changes were not detected in Skn-1a^−/−^ mice (Figures 2C, D). At the end of the observation period, blood total GLP-1 levels were significantly increased by EGCG administration (Figure 2E). Additionally, blood insulin concentrations showed a tendency to increase in the EGCG group (Figure 2F). As shown in Figures 5E, F, these changes were not observed in Skn-1a^−/−^ mice.

### 3.2 Chronic EGCG treatment reduces weight gain and caloric intake

**Figure 3:**
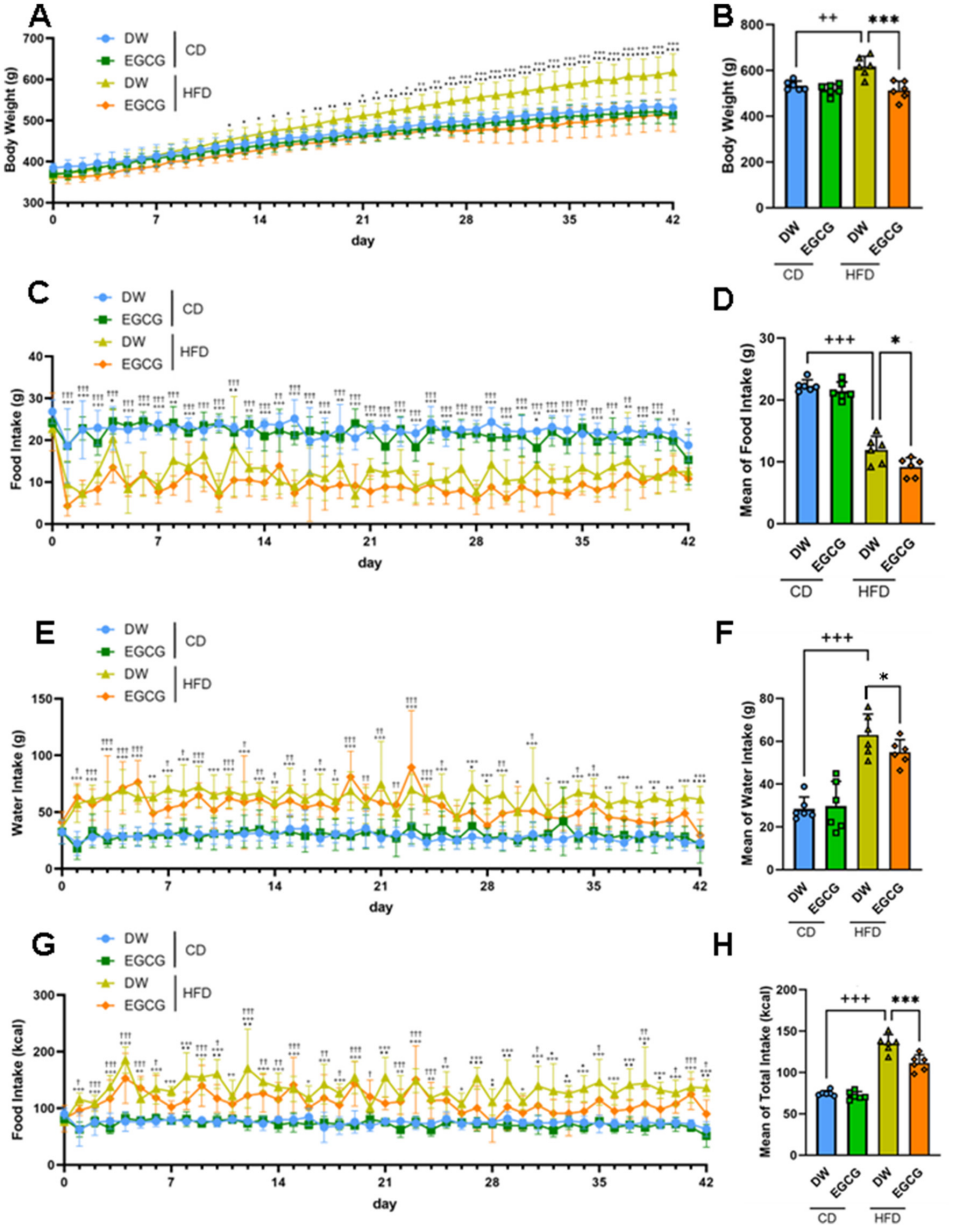
Effect of a 6-week repeated oral administration of EGCG on body weight, food intake, water intake, and calorie intake in rats fed a normal or high-fat diet (with 30% sucrose in drinking water). Values are expressed as mean ± SD (*n* = 6 per group). Statistical analyses for **(A, C, D, E, G)** were performed using two-way ANOVA followed by Tukey's multiple comparisons test. For **(B, D, F, H)**, one-way ANOVA followed by Bonferroni's multiple comparisons test was used. Statistical significance compared with CD-DW: +*p* < 0.05, ++*p* < 0.01, +++*p* < 0.001; Compared with HFSD-DW: *p < 0.05, **p < 0.01, ***p < 0.001; Compared with CD-EGCG:^†^ *p* < 0.05,^†^^†^ *p* < 0.01,^†^^†^^†^ *p* < 0.001.

In the HFSD-DW group, there was a significant increase in body weight, atrophy of the heart, soleus, and extensor digitorum longus, and a significant increase in epididymal adipose compared to the CD-DW group, but no such changes were observed in the HFSD-EGCG group (Table 1). Figure 3 shows the time-dependent changes in body weight (Figure 3A), food intake (Figure 3C), fluid intake (Figure 3E), and calorie intake (Figure 3G) of rats orally administered either distilled water (DW) or 100 mg/kg EGCG while being fed a control diet (CD) or a high-fat/high-sucrose diet (HFSD) for 6 weeks. Additionally, body weight after 6 weeks (Figure 3B), total food intake (Figure 3D), total fluid intake (Figure 3F), and total calorie intake (Figure 3H) during the experimental period are presented. Compared with the CD-DW group, the HFSD-DW group showed a significant increase in body weight starting from day 12 of the treatment, whereas this change was not observed in the other experimental groups (Figures 3A, B). Food intake was markedly lower in the HFSD groups than in the CD groups (Figures 3C, D). Total food intake was significantly lower in the HFSD-EGCG group compared to the HFSD-DW group (Figure 3D). While the intake of water or 30% sucrose was markedly higher in the HFSD groups than in the CD groups (Figures 3E, F), total fluid intake was significantly lower in the HFSD-EGCG group than in the HFSD-DW group (Figure 3F). Calorie intake was significantly higher in the HFSD groups than in the CD groups (Figures 3G, H). Total calorie intake was significantly lower in the HFSD-EGCG group than in the HFSD-DW group (Figure 3H).

**Table 1:** Body weight and tissue weight per of body weight at the end of the feeding period.

	CD		HFSD	
	DW	EGCG	DW	EGCG
Body weight (final, g)	513.501 ± 3.38	514.801 ± 7.40	611.331 ± 6.33^+++^	514.401 ± 1.41
Heart (g/kg)	2.720 ± 0.21	2.760 ± 0.40	2.280 ± 0.12^+^	2.610 ± 0.28
Kidney (g/kg)	5.810 ± 0.38	6.170 ± 0.64	5.420 ± 0.37	6.270 ± 0.59
Liver (g/kg)	24.141 ± 0.43	24.141 ± 0.43	23.731 ± 0.15	23.731 ± 0.15
Spleen (g/kg)	1.900 ± 0.56	1.890 ± 0.14	1.770 ± 0.28	2.110 ± 0.62
Adrenal grande (g/kg)	0.150 ± 0.04	0.170 ± 0.03	0.130 ± 0.02	0.150 ± 0.02
Brown fat (g/kg)	0.660 ± 0.22	0.780 ± 0.21	0.890 ± 0.17	1.080 ± 0.21
Total white adipose (g/kg)	94.886 ± 0.47	65.401 ± 9.18	112.482 ± 2.11	79.929 ± 0.85
Perirenal adipose (g/kg)	29.362 ± 0.56	23.388 ± 0.70	45.671 ± 0.00	19.013 ± 0.96
Epididymal adipose (g/kg)	25.932 ± 0.92	24.716 ± 0.33	36.665 ± 0.66^+^	28.083 ± 0.38
Mesenteric adipose (g/kg)	18.532 ± 0.03	17.454 ± 0.86	28.727 ± 0.20	19.013 ± 0.96
Inguinal adipose (g/kg)	1.180 ± 0.37	1.200 ± 0.35	1.270 ± 0.27	1.120 ± 0.19
Soleus (g/kg)	0.460 ± 0.05	0.450 ± 0.08	0.320 ± 0.04^++^	0.420 ± 0.03
Gastrocnemius (g/kg)	2.970 ± 0.56	3.501 ± 0.26	3.900 ± 0.27	4.490 ± 0.86
Extensor digitorum longus (g/kg)	0.450 ± 0.05	0.460 ± 0.04	0.360 ± 0.04^+^	0.430 ± 0.07
Tibialis anterior (g/kg)	1.720 ± 0.13	2.040 ± 0.37	2.110 ± 0.23	2.470 ± 0.18

Each value represents mean and SD (*n* = 6). Significantly difference from CD-DW, +; *p* < 0.05, ++; *p* < 0.01, +++; *p* < 0.001. Significantly difference from HFSD-DW, +; *p* < 0.05, ++; *p* < 0.01, +++; *p* < 0.001.

**Figure 4:**
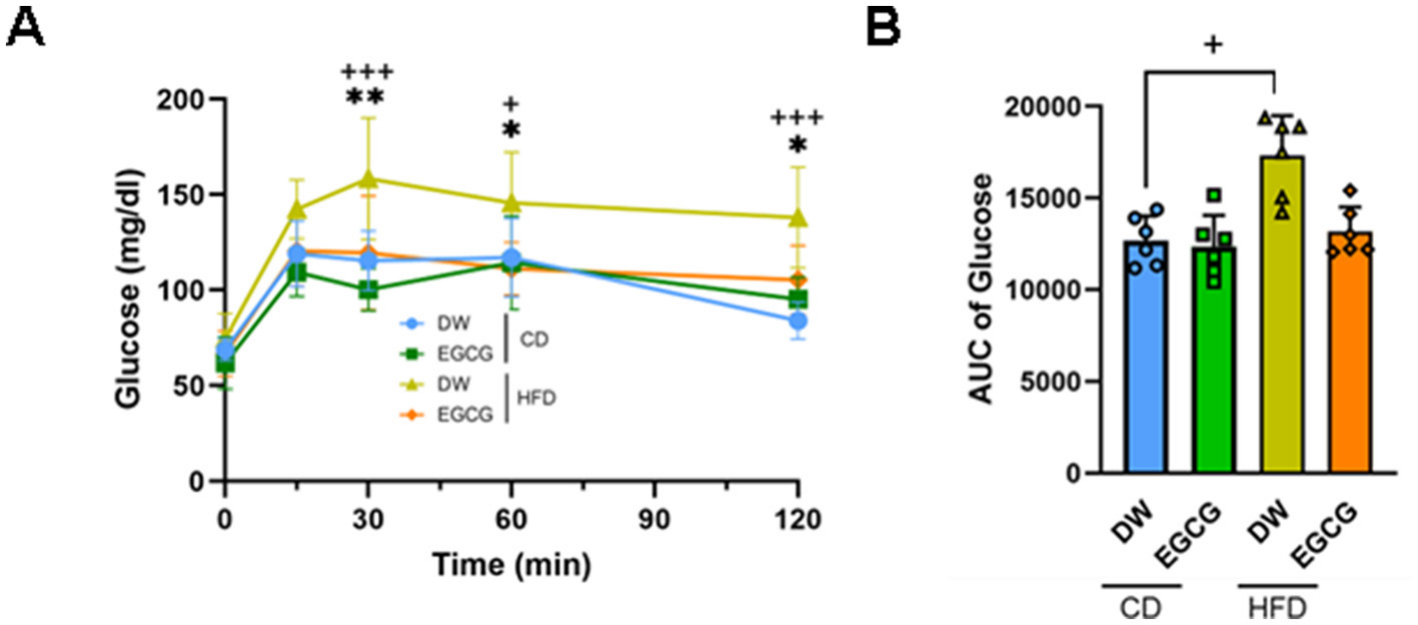
Effect of a 6-week repeated oral administration of EGCG on glucose tolerance, assessed via the intraperitoneal glucose tolerance test (IPGTT) in rats fed a normal or high-fat diet (with 30% sucrose in drinking water). Changes in blood glucose **(A)** and the area under the curve (AUC) **(B)** in rats fed a normal or high-fat diet (with 30% sucrose in drinking water). Values are expressed as mean ± SD (*n* = 6 per group). Statistical analyses for A were performed using two-way ANOVA, followed by Tukey's multiple comparisons test. For B, one-way ANOVA followed by Bonferroni's multiple comparisons test was used. Statistical significance compared with CD-DW: +*p* < 0.05, +++*p* < 0.001; compared with HFSD-DW; *p < 0.05, **p < 0.01.

The results of IPGTT conducted at the end of the feeding period are shown in Figure 4. In the HFSD-DW group, blood glucose levels at 30–120 min after glucose administration were significantly higher than those in the CD-DW group (Figure 4A), and the area under the curve (AUC) was also significantly elevated (Figure 4B). Conversely, in the HFSD-EGCG group, the blood glucose profile was like that of the CD groups, with no significant change in the AUC.

### 3.3 EGCG attenuates neuroinflammation

**Figure 5:**
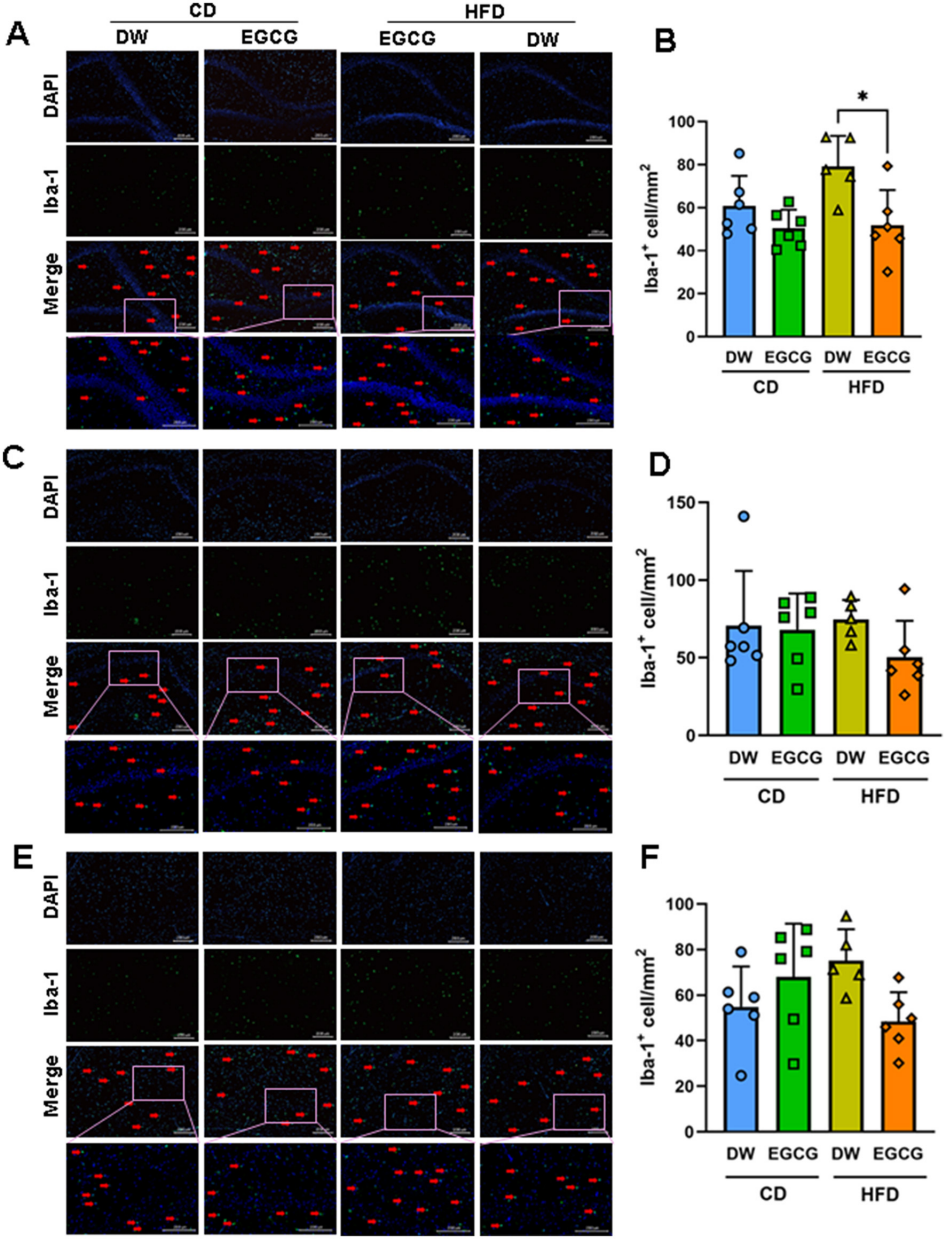
Effect of a 6-week repeated oral administration of EGCG on iba-1 expression in the hippocampus in rats fed a normal or high-fat diet (with 30% sucrose in drinking water). Typical microscopic images of the dentate gyrus **(A)**, CA1 region **(C)**, and CA3 region **(E)** are shown, and the number of ibA-1-positive cells per frame is shown in **(B, D, F)**, respectively. The red arrow indicates a positive cell. Values are expressed as mean ± SD (*n* = 6 per group). Statistical analyses for **(B, D, E)** were performed using one-way ANOVA followed by Bonferroni's multiple comparisons test. Statistical significance compared with CD-DW: HFSD-DW: * *p* < 0.05.

Immunostained images of the hippocampal dentate gyrus (DG), CA1, and CA3 regions are presented in Figure 5. In the DG, the number of Iba-1-positive cells exhibited an increasing trend in the HFSD-DW group compared to the CD-DW group (Figure 5A). Notably, EGCG administration significantly decreased the number of Iba-1-positive cells in the HFSD-EGCG group compared to the HFSD-DW group. No significant changes were observed in the CA1 or CA3 regions.

### 3.4 EGCG attenuates adipocyte hypertrophy

**Figure 6:**
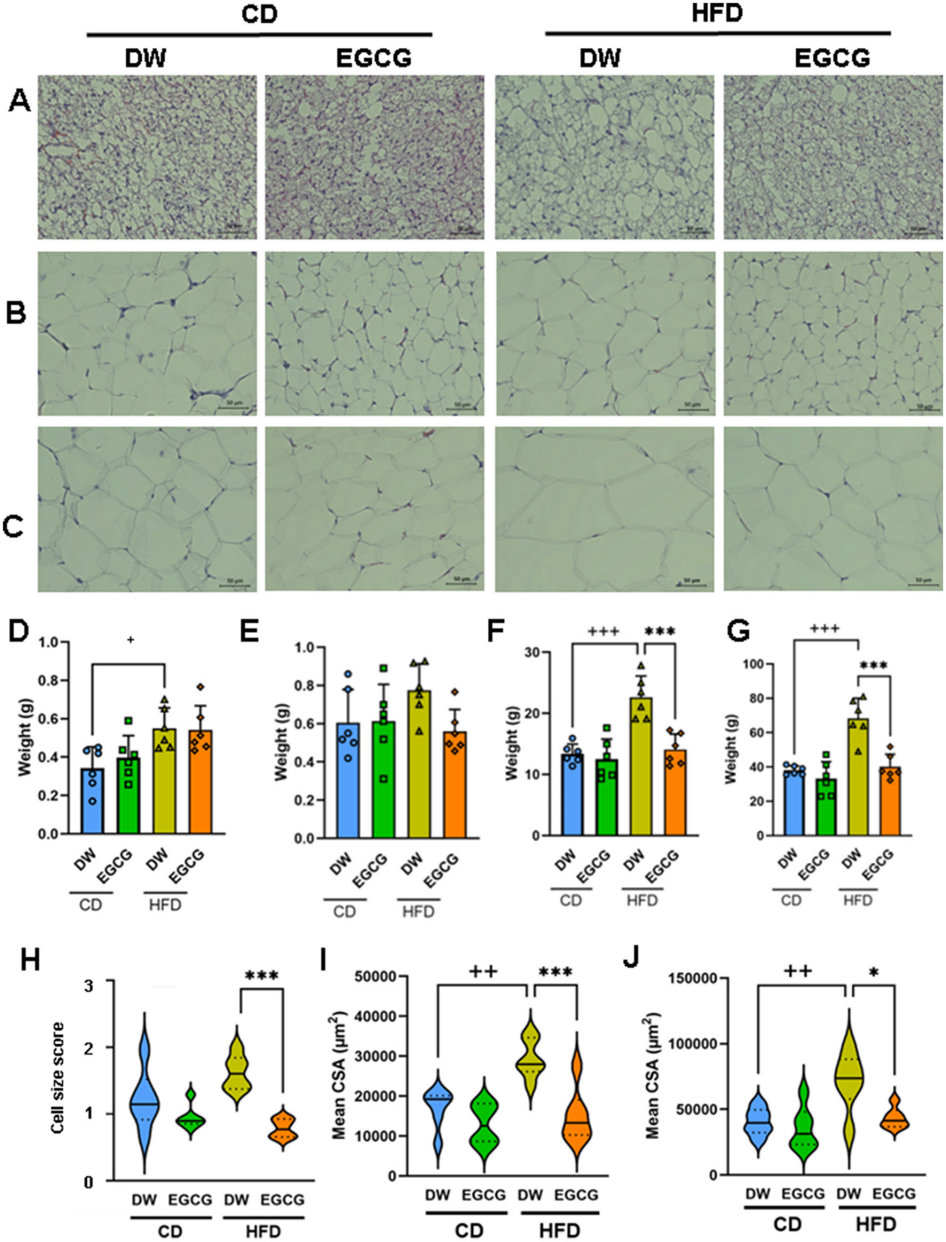
Effect of a 6-week repeated oral administration of EGCG on adipose tissue in rats fed a normal or high-fat diet (with 30% sucrose in drinking water). Typical microscopic images of the brown adipose **(A)**, inguinal adipose **(B)**, and epididymal adipose **(C)** are shown, and the weight is shown in **(D–F)**, respectively. The weight of total white adipose (perirenal, epididymal, mesenteric and inguinal adipose) is represented in **(G)**. Cell size score of brown fat was shown in H. Mean cross section area (CSA) of inguinal adipose **(I)** and epididymal adipose **(J)** are shown in **(I, J)**. Values are expressed as mean ± SD (*n* = 6 per group). Statistical analyses for **(D–G)** were performed using one-way ANOVA followed by Bonferroni's multiple comparisons test. For **(H–J)**, Dunn's multiple comparisons test was performed following the Kruskal–Wallis test. Statistical significance compared with CD-DW: +*p* < 0.05, ++*p* < 0.01, +++*p* < 0.001; Compared with HFSD-DW: * *p* < 0.05, *** *p* < 0.001.

Histopathological analysis of adipose tissue revealed that the mass of brown, epididymal, and total white adipose tissue significantly increased in the HFSD-DW group compared to the CD-DW group (Figures 6A–G). EGCG treatment significantly inhibited these increases in epididymal and total white fat mass. Furthermore, the cross-sectional area (CSA) of inguinal and epididymal adipose tissues was significantly increased by HFSD, but these changes were markedly suppressed by EGCG (Figures 6H–J), with the decrease of cell size score of BAT.

### 3.5 EGCG induces skeletal muscle hypertrophy independent of diet

**Figure 7:**
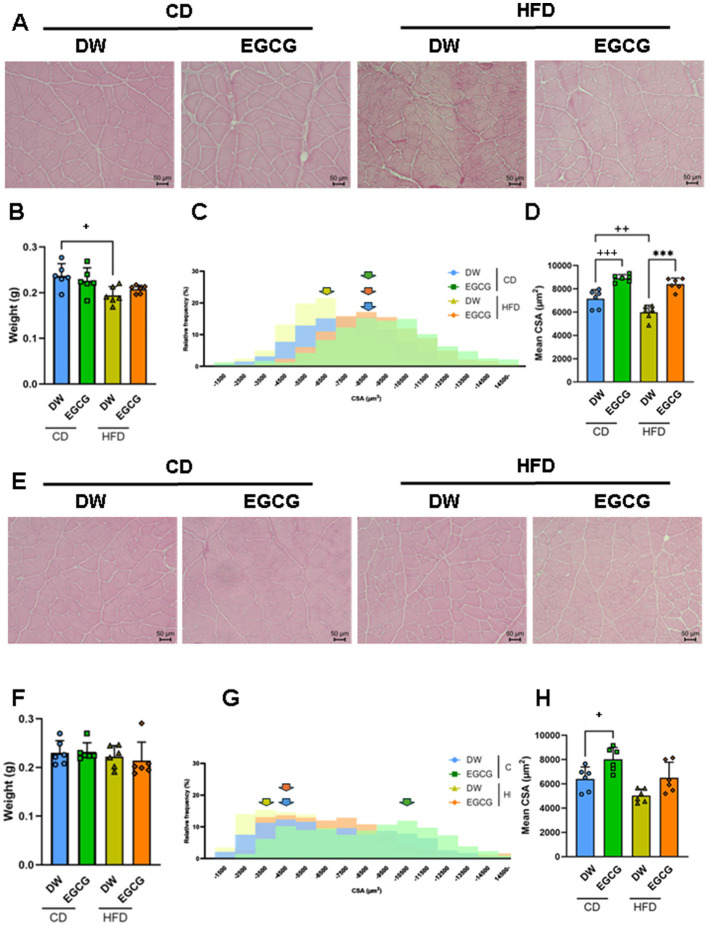
Effect of a 6-week repeated oral administration of EGCG on skeletal muscle in rats fed a normal or high-fat diet (with 30% sucrose in drinking water). Typical microscopic images, weight, cell size distribution (the arrow indicates the peak), and mean cross-sectional area (CSA) of the soleus (**A–D)** and extensor digitorum longus **(E–H)** are represented. Values are expressed as mean ± SD (*n* = 6 per group). Statistical analyses for **(B, D, F, H)** were performed using one-way ANOVA followed by Bonferroni's multiple comparisons test. Statistical significance compared with CD-DW: + *p* < 0.05, ++*p* < 0.01, +++*p* < 0.001; Compared with HFSD-DW: *** *p* < 0.001.

Regardless of the diet type, repeated EGCG intake brought about substantial alterations in skeletal muscle characteristics (Figure 7). In the soleus muscle (slow-twitch), both muscle weight and mean CSA were significantly lower in the HFSD-DW group than in the CD-DW group (Figures 7B, D). Similarly, in the extensor digitorum longus (EDL) muscle (fast-twitch), the mean CSA tended to decrease under HFSD (Figure 7H). In both muscle types, the CSA distribution peaks shifted toward smaller sizes in the HFSD-DW group but were restored to higher values in the HFSD-EGCG group (Figures 7C, G). Importantly, EGCG administration increased the mean CSA in both muscles irrespective of the diet (Figures 7D, H). This series of findings suggests the existence of a metabolic mechanism independent of food intake suppression.

## 4 Discussion

### 4.1 T2R-mediated regulation of glucose homeostasis

T2R receptors play a critical role in survival by enabling animals to avoid harmful substances through the detection of plant-derived toxins. While humans typically tolerate mild bitterness in safe foods (32), recent studies have revealed that T2R interactions do more than just facilitate toxin avoidance; they regulate metabolic systems via gastrointestinal hormone secretion and enhance biological defense mechanisms (33). In this study, we utilized DB as a positive control and EGCG, a gallated catechin with a distinct bitter and astringent profile (22), to investigate the role of T2Rs in glucose regulation.

By employing the Skn-1a^−/−^ mouse model—which lacks Trpm5-positive chemosensory cells due to the absence of the key transcription factor Pou2f3 (34, 35)—we conclusively demonstrated that the acute glucose-lowering effects and GLP-1 elevation induced by DB and EGCG are Skn-1a-dependent (Figures 1, 2). Our findings that EGCG triggers GLP-1 secretion in a Skn-1a-dependent manner align with and extend recent observations that certain dietary polyphenols can modulate gastrointestinal hormone production via chemosensory pathways. These findings suggest that T2Rs function as essential sensors that trigger metabolic responses immediately upon oral or gastric exposure, independent of systemic absorption. This effect, initiated by the luminal sensing of EGCG by Skn-1a-dependent chemosensory cells, occurs independently of systemic absorption. Specifically, luminal exposure in the stomach and proximal small intestine allows EGCG to act as a signaling molecule that activates the gut-brain axis, triggering hormone release (e.g., GLP-1) before the compound reaches the systemic circulation. This non-absorptive mechanism explains how EGCG can exert profound physiological effects despite its notoriously low bioavailability.

Furthermore, it is noteworthy that Skn-1a^−/−^ mice exhibited significantly different basal levels of GLP-1 and insulin compared to wild-type mice even prior to administration (Figures 1E, F). These observations suggest that the Skn-1a-dependent chemosensory system is not merely an “on-demand” responder to acute dietary stimuli, but rather a fundamental regulator of basal metabolic tone. The absence of these chemosensory cells may lead to a chronic disruption in the gut-brain-pancreas axis, potentially pre-configuring the animal to a state of metabolic dysfunction.

In this study, we employed an EGCG dose of 100 mg/kg in mice, which corresponds to an HED of 567 mg/day for a 70 kg human. This dosage is critically achievable through standard human dietary practices or supplementation. For instance, given that a typical cup of green tea contains approximately 50–100 mg of EGCG, the calculated HED is equivalent to consuming 6–10 cups of green tea daily. While high, this level of intake is common in regular tea-consuming populations. Furthermore, 500 mg is a standard dose in commercially available EGCG supplements, making our findings practically applicable to human health strategies.

### 4.2 Appetite suppression and metabolic improvement via the gut-brain axis

Chronic activation of T2Rs has been shown to suppress appetite by modulating the secretion of anorexigenic hormones such as CCK, GLP-1, and PYY (36, 37). Specifically, PYY secreted from L-cells binds to Y2 receptors on vagal afferent fibers, transmitting satiety signals to the hypothalamic arcuate nucleus (ARC) via the nucleus tractus solitarius (NTS) (38, 39). This pathway subsequently activates the POMC/CART neurons while inhibiting the NPY/AgRP hunger system (40). In our chronic rat study, we observed a distinct shift in dietary behavior among the HFSD-fed animals. These rats decreased their solid food intake but significantly increased their consumption of the 30% sucrose solution (Figure 3). This phenomenon is consistent with previous findings that laboratory animals prioritize highly palatable liquid calories over solid nutrition. Crucially, while the total caloric intake remained higher in the HFSD-DW control group—driving the observed weight gain—EGCG administration significantly suppressed the excessive intake of this sucrose solution. This suggests that EGCG-mediated sensory signaling may specifically attenuate reward-based feeding or enhance the satiety signals (such as GLP-1 and PYY) that regulate the intake of palatable energy sources.

### 4.3 Distinct roles of bitter and astringent signaling: from glucose regulation to exercise-mimetic effects

A particularly striking observation in this study was that EGCG induced skeletal muscle hypertrophy and suppressed hippocampal neuroinflammation (indicated by reduced Iba-1 expression) regardless of the dietary composition (Figures 5, 7). These findings suggest a metabolic mechanism that extends beyond simple caloric restriction. We propose that these systemic effects for skeletal muscle or brain are driven by the sensory characteristics of EGCG specifically its astringency, as distinct from its T2R-mediated bitterness.

While astringency was once thought to be a purely mechanical sensation, emerging evidence suggests the involvement of Transient Receptor Potential (TRP) channels in sensory neurons (41, 42). We hypothesize that these stimuli activate the locus coeruleus-noradrenergic (LC-NE) and hypothalamic CRH neurons, thereby enhancing sympathetic nervous system (SNS) activity via the sympathetic-adrenal medullary (SAM) axis (43). The resulting release of noradrenaline and adrenaline coordinates fatty acid oxidation in adipose tissue (via β3-receptors and UCP-1 browning) and protein synthesis in skeletal muscle (via β2-receptors and the PKA/AMPK pathway) (44, 45). Consequently, chronic EGCG-induced SAM axis activation provides physiological benefits that mimic the effects of physical exercise (46).

### 4.4 Causality of neurological effects: sensory signaling vs. metabolic consequences

To critically evaluate the causality of the observed neuroprotection, we must weigh direct sensory signaling against indirect metabolic benefits. The total abolition of both GLP-1 elevation and neuroinflammatory suppression in Skn-1a^−/−^ mice provides strong evidence that initial sensory signaling is the “master switch.” While improved peripheral glucose homeostasis and reduced systemic inflammation naturally lower metabolic stress on the CNS, these are likely secondary consequences initiated by the gut-brain axis trigger. Furthermore, given EGCG's low bioavailability, its ability to cross the blood-brain barrier for direct action is likely subordinate to the robust, immediate signaling initiated by gut chemosensors.

### 4.5 Limitations and perspectives

Despite these novel insights into sensory-driven regulation, several limitations remain. Although we emphasize a sensory-mediated mechanism, the potential contributions of EGCG metabolites or modifications in the gut microbiota cannot be entirely dismissed. While we established a link between EGCG and T2Rs for glucose tolerance, the direct causal involvement of specific receptors in muscle hypertrophy and neuroprotection requires further validation. The study showed that skeletal muscle CSA increased and fiber size changed after chronic EGCG administration, aligning with the activation of the sympathetic-adrenal medullary (SAM) axis and subsequent β2-adrenergic signaling. However, muscle CSA primarily serves as a morphological proxy for hypertrophy. While EGCG promoted hypertrophy-like changes in two muscles, grip strength, treadmill endurance and muscle force were not assessed. Therefore, while our data demonstrates a clear structural response to EGCG-mediated sensory signaling, the term “exercise-mimetic” should be interpreted with caution. Nevertheless, the observation that EGCG can induce significant muscle fiber remodeling regardless of dietary composition (CD vs. HFSD) provides a promising foundation for developing “Sensory Nutrition” strategies to counteract sarcopenia and metabolic decline in sedentary populations. Future clinical trials are necessary to confirm whether these “Sensory Nutrition” pathways are similarly potent in humans, accounting for interspecies differences in receptor sensitivity.

## 5 Conclusion

In conclusion, EGCG enhances glucose tolerance through bitter taste receptors and effectively mitigates the central and peripheral impairments induced by high-energy diets. Notably, EGCG suppresses visceral adiposity and hippocampal neuroinflammation while promoting skeletal muscle hypertrophy, regardless of dietary composition. These physiological benefits are achieved through a mechanism distinct from simple caloric restriction. Our data supports a model where EGCG-induced astringency acts as a potent sensory stimulus that activates the SAM axis. The resulting chronic elevation of catecholamines likely drives the “exercise-mimetic” effects observed in adipose and muscle tissues. These findings underscore the potential of “Sensory Nutrition,” a paradigm that leverages food's functional properties to regulate the autonomic nervous system via sensory pathways. EGCG and similar astringent compounds represent promising candidates for next-generation functional foods designed to prevent obesity-related cognitive decline and sarcopenia.

## Data Availability

The original contributions presented in the study are included in the article/Supplementary material, further inquiries can be directed to the corresponding author.
