# Supplementary material for: Sensory signaling mediates the systemic metabolic and neurological effects of epigallocatechin gallate
Source: Front Nutr. 2026 Jul 3;13:1863138. doi: 10.3389/fnut.2026.1863138 (PMC13375716; doi:10.3389/fnut.2026.1863138)
Supplement: Supplementary file 1 [file Data_Sheet_1.PDF]

Supplemental information

Sensory Signaling Mediates the Systemic Metabolic and Neurological Effects of Epigallocatechin Gallate

Yamato Yoshida[1*], Naoki Iida[1], Kenshin Iwasa[1], Akuru Saito[1], Kenta Aso[2], Yasuyuki Fujii[3,4], Sergio Modafferi[5], Vittorio Calabrese[5], Makoto Ohmoto[6], Keiko Abe[7], Naomi Osakabe[1#]

Stabel 1 The alteration of blood glucose after a single oral administration of different level

blood glucose (mg/dL)	vehivle			EGCG 10mg/kg			
0	120.1	±	13.5	132.1	±	24.4	
15	184.8	±	14.9	199.3	±	21.4	
30	232.4	±	35.8	258.9	±	23.9	
60	230.9	±	57.7	265.3	±	71.3	
120	279.4	±	139.9	160.9		16.9	
**blood glucose (mg/dL)**	**vehivle**			**EGCG 50mg/kg**			
0	73.6	±	7.7	83.6	±	27.5	
15	116.7	±	34.8	121.4	±	41.9	
30	139.5	±	40.7	139.0	±	59.7	
60	146.6	±	45.0	120.6	±	60.9	
120	143.5	±	31.5	104.8	±	44.6	#
**blood glucose (mg/dL)**	**vehivle**			**EGCG 100mg/kg**			
0	106.9	±	21.9	94.1	±	17.3	
15	288.6	±	45.0	251.4	±	44.4	
30	353.0	±	82.7	274.8	±	34.4	*
60	294.7	±	64.7	245.1	±	44.1	#
120	253.3	±	106.9	181.7	±	37.9	#

Each value represents the mean and SD. #p<0.1,*p<0.05)unpaired t-test)

Stabel2 Composition of a high-fat diet (HFD®)

Ingredient	Amount (%)
Milk Casein	24.50
Egg White Powder	5.00
L-Cystine	0.43
Powdered Beef Tallow	16.72
Safflower Oil (High-Oleic)	20.00
Crystalline Cellulose	5.50
Maltodextrin	7.65
Lactose	6.69
Sucrose	6.75
AIN-93 Vitamin Mix	1.40
AIN-93G Mineral Mix	5.00
Choline Bitartrate	0.36
t-Butylhydroquinone (TBHQ)	0.00
Total	100.00
